# Smart home for elderly care: development and challenges in China

**DOI:** 10.1186/s12877-020-01737-y

**Published:** 2020-09-03

**Authors:** Quan Zhang, Meiyu Li, Yijin Wu

**Affiliations:** 1 Ocean University of China, 238 Songling Road, Qingdao, 266100 PR China; 2 China University of Petroleum (East China), 66 Changjiangxi Road, Qingdao, 266580 PR China; 3 School of Translation Studies/Center for Medical Humanities in the Developing World, Qufu Normal University, No. 80, Yantai Road, Donggang District, Rizhao, 287600 Shandong PR China

**Keywords:** Smart home, Elderly care, China

## Abstract

**Background:**

China’s smart home for elderly care emerged in 2008, and had went through four developmental stages which consists of seed stage, start-up stage, development stage and popularization stage.

**Main text:**

The status quo and development of smart home for elderly care in China is reviewed, and suggestions are provided on how to further develop China’s smart home for elderly care. The focus of China’s policies on smart home for elderly care were different during those four developmental stages. Compared with Western countries, China’s smart home for elderly care is a policy-driven product rather than technology-driven or demand-driven one. In addition, it is quasi-public goods rather than private goods. These unique characteristics of China’s smart home for elderly care not only become the driving force of its rapid development, but also bring many challenges to its development, such as the insufficient demand, the disorderly development, and the waste of public and private resources.

**Conclusions:**

Although great progress has been made in China’s smart home care, much efforts are still needed to further advance its development. The technical standards for the elderly care services should be formulated as soon as possible and the existing public and private smart home for elderly care platforms should be combined. Enterprises involved in smart home care services should be encouraged to develop new technologies to reduce the cost of products and services provided by smart home for elderly care.

## Background

After the second world war, the increasing ageing population has brought huge pressure on the economic and social life of all countries [1]. The demographic structure of the world will change significantly in the next 40 years, which requires each country to shift from the welfare model to individual model. In this sense, family will become the main place for caring the elderly [2]. In addition, modern medicine brings about an increase in life expectancy, which leads to an increasing demand for the elderly care services [3]. Due to social transformation and job mobility, it is increasingly difficult for the elderly to rely on their family members to take care of them. In addition, a limited number of nursing staff in China cannot meet the rising demand for the elderly care services. Therefore, how to meet the demands of home-based elderly people for medical care and nursing services has become a key issue for the development of the elderly care services in the world.

In recent years, the fourth scientific and technological revolution characterized by Internet of Things (IoTs), Information Technology, Big Data and Cloud Computing has greatly promoted the development of the elderly care services. A number of emerging technologies have been used to facilitate the development of aging-in-place, among which the smart home technology for elderly care is an important one [4]. Smart home for elderly care is committed to providing various services to meet the demands of the elderly such as safety, independence, health and assistance at a low cost [5]. Thus, smart home care plays a significant role in caring for the elderly [6].

Smart home has developed in both developed and developing countries. Smart home was first proposed by the American Association of Builders in 1984, which refers to houses with intelligent interactive technology [7]. Smart home has made significant progress since Internet of Things, Information Technology, Big Data and Cloud Computing were involved. It can not only record, store and analyse the users’ habits and needs [8] and interact with users in various ways such as sound, light, position and image [9], but also link external resources through information technology to provide users with various services such as health, assistance, security, education, and entertainment [10]. Smart home for the elderly people concerns the application of smart home to the elderly care, which pays more attention to the needs of safety, independence and health of the elderly. In different countries and regions, smart home for the elderly people has different names. For instance, in the United Kingdom and the United States of America it is called smart home for the elderly or smart home for seniors, which means a residence equipped with modern technology that could enhance safety of the elderly and monitors their health conditions [11]. In Sweden, it is called e-Home care, which involves the application of Information and Communication Technology (ICT) to home-care. E-home care services include monitoring, reminders, information services and social interaction [12]. In German, it is called Ambient Assisted Living (AAL), which refers to intelligentizing all kinds of household appliances on a scalable intelligence technology platform through modern induction transmission devices. AAL could make an immediate response to the elder’s physical condition and home environment [13].

In 2015, China’s prime minister Li Keqiang put forward the “Internet plus” plan in the *Report on the Work of the Government.* Immediately after this, the National Development and Reform Commission (NDRC) of China in collaboration with other departments issued a notice, which proposes to apply the “Internet plus” plan to elderly care. The essence of “Internet plus” plan concerns the integration of the Internet technologies into various aspects of economics and society in order to form a new pattern of economics and social development. Specifically, “Internet plus” plan consists of internet plus manufacturing, internet plus modern agriculture, internet plus smart energy and internet plus public services and so on [14]. As the core content of internet plus the elderly care services, smart home for elderly people have become the key development direction of China government.

Smart home for the elderly in China is generally the same as the definition of smart home for the elderly in Western countries, which refers to the application of Internet of things, information technology, big data, cloud computing and other technologies to elderly care in order to provide the elderly with smart care or smart home environment, meet their needs for healthy and independent life, and finally improve their physical and mental health and quality of life [15]. As the largest developing country in the world, what are China’s policies on smart home for elderly care? What are the achievements of and challenges for China’s smart home for elderly care? How China responded to these challenges? By answering these questions, we could have a better understanding of development and challenges of China’s smart home for elderly care.

## Main text

In this study, the status quo and development of smart home for elderly care in China is reviewed. China’s smart home for elderly care emerged in 2008, and had went through four development stages which consists of seed stage, start-up stage, development stage and popularization stage. These four developmental stages of smart home for elderly care are reviewed and discussed in detail. In addition, the characteristics of smart home for elderly care in China are elaborated on. Furthermore, the challenges for the development of smart home for elderly care in China are presented in detail. Finally, suggestions are provided on how to further develop China’s smart home for elderly care.

### Policies and development of smart home for elderly Care in China

#### Seed stage (2008–2011)

In 2008, China National Working Commission on Ageing (CNWCA) along with other departments jointly issued *Opinions on Comprehensively Promoting Home-based Elderly Care Services*, proposing that China should establish various forms of services such as hotline and emergency rescue system in communities which could build convenient and effective service information system for the elderly. In 2011, General Office of the State Council issued *Development Plan of Social Elderly Care Service System (2011–2015)*, stipulating that China would establish community-based elderly care platform by using hotline, information network, and community-based calling system, which could provide daily care, emergency assistance and other caring services for the elderly. These two documents issued above started China’s smart home for elderly care. During that period, a number of cities in China including Beijing, Shanghai and Nanjing successively started up 12,349 home-based elderly care service hotline. The establishment of 12,349 hotline was a great achievement of China’s smart home for elderly care.

#### Start-up stage (2012–2014)

In June 2012, China’s 427th Xiangshan Scientific Conference was held in Beijing, which proposed a four-tier service framework for smart home for elderly care. This proposal was adopted by the central government. In the following year, the Ministry of Civil Affairs together with National Development and Reform Commission (NDRC) issued *Notice on Comprehensive Reform Pilot of Elderly Care Service Industry*, stating that technologies such as the Internet and IoTs could be used to improve the management of elderly care. In 2013, China’s State Council issued *Several Opinions on Speeding up the Development of Elderly Care Service Industry*, stating that local governments should encourage the enterprises and institutions to use new technologies such as internet and IoTs to explore new ways for providing elderly care, and build elderly care network platform such as emergency calls, health consultation, and service booking to provide convenient home care services. In 2014, the Ministry of Civil Affairs launched a national-level demonstration project for the application of smart home for elderly care in seven nursing institutions. This project applies the IoTs to carry out caring services such as falling detection, sleep monitoring and self-service physical examination in nursing institutions [16]. In the same year, Shanghai issued *Guiding Opinions on Promoting the Pilot Construction of Livable Communities for the Elderly*, and set up smart care centers in 40 pilot communities to provid*e* smart home care services such as security monitoring, emergency assistance and daily care for the elderly [17]. These two pilot projects showed that China’s smart home for elderly care was put into practice.

#### Development stage (2015–2016)

In 2015, China’s prime minister Li Keqiang put forward “Internet plus” plan in the *Report on the Work of the Government*. Then, National Development and Reform Commission (NDRC) of China along with other government agencies issued *Instructions to Promoting Internet plus Plan*, which offers detailed guides on how to apply the“Internet plus” plan to elderly care. For instance, health service provider should be encouraged to build public information platform based on the technologies such as cloud computing, big data, which could provide long-term tracking, health condition forecasting and other individualized health management service. Also, building community-based elderly care information platform could provide such smart home for elderly care as nursing care, health management and rehabilitation care for the elderly. This document provides general guidance for the development of smart home for elderly care in China. In 2016, the Ministry of Civil Affairs along with the Ministry of Finance issued *Notice on the Pilot Reform of Home-based Elderly Care Conducted by the Central Budget*, stating that China will explore multiple ways to strengthen the relationship between supply and demand, and provide high quality services with low costs for the elderly. In 2016, the General Office of the State Council issued *Several Opinions on Opening up the Elderly Care Service Market and Improving the Quality of Elder Care Services,* which allows private aged care providers access to smart home services for the elderly. Driven by the national policies above, almost all provinces in China issued local policies to promote smart home for elderly care by the end of 2016.

#### Popularization stage (2017–2019)

From 2017 to 2019, China issued three policy documents to comprehensively promote smart home for elderly care. In 2017, the State Council issued the *The 13th Five-year Plan for the Aging Development and Elderly Care Services*, stating that China would establish community-based elderly care service information platform, service order system and emergency rescue mechanism which could provide the elderly with various kinds of caring services such as food feeding, walking aids, and daily nursing care. In 2017, the National Health and Family Planning Commission (NHFPC) and other government agencies issued *The 13th Five-year Plan for Healthy Aging (2016–2020)*, proposing that NHFPC would make full use of the Internet, IoTs, Big Data and other information technologies to explore the new models of care services for the elderly, and establish home and community-based smart health care pilot projects. At the same time, the NHFPC would build smart health and caring service platform, where medical authorities and elderly care service providers could provide health guidance, chronic disease management, safety monitoring and other caring services for the elderly. In 2017, the Ministry of Industry and Information Technology (MIIT) and the Ministry of Civil Affairs issued *Action Plan for the Development of Smart Health and Elderly Care Services (2017–2020)*, which is the first specialized national policy on smart home for elderly care in China. This policy not only clarified the objectives and overall planning for the development of smart home for elderly care, but also put forward specific action plans on key issues such as technologies research, service popularization, the establishment of the smart care platform and the formulation of the industry standard. Driven by this national policy, smart home for elderly care has mushroomed in China. The main policy documents on China’s smart home for elderly care are listed in Table 1.

**Table 1:** Main policy documents of China’s smart home for elderly care

Stage	Policy document(s)	Department	Time	Main content
Seed stage (2008–2011)	*Opinions on Comprehensively Promoting Home-based Elderly Care Services*	National Working Commission on Ageing.etc	2008	To establish elderly care hotline, emergency rescue system and service information platform in community
	*Construction Plan of Social Elderly Care Service System (2011–2015)*	General Office of the State Council	2011	To construct community-based elderly care platform using hotline, information network, community-based calling system
Start-up stage (2012–2014)	*Notice on Comprehensive Reform Pilot of Elderly Care Service Industry*	Ministry of Civil Affairs, National Development and Reform commission	2013	To use internet, IoTs and other technologies to improve the managemental and informationalized level of elderly care services
	*Several Opinions on Speeding up the Development of Elderly Care Service Industry*	State Council of China	2013	To build smart platform for community to provide emergency call, health consultation and other elderly services
Development stage (2015–2016)	*Instruction to Promoting Internet plus Plan*	National Development and Reform commission.etc	2015	To encourage health service organizations to use cloud computing, big data and other technologies to build public information platforms and provide personalized and long-term health management services
	*Notice on the Pilot Reform of Home-based Elderly Care Conducted by the Central Budget*	Ministry of Civil Affairs, Ministry of Finance	2016	To develop smart home care technology, enrich smart homecare modes and reduce the cost of smart homecare for the elderly
	*Several Opinions on Fully Opening up the Elderly Care Service Market and Improving Elderly Care Service’s Quality*	General Office of the State Council	2016	To allow and encourage private capital to provide smart home care services for the elderly
Popularization stage (2017–2019)	*The 13th Five-year Plan for the Aging Development and Elderly Care Service System Construction*	State Council of China	2017	To establish information platform for home-based community elderly care services focusing on disabled, solitary and empty nest elderly
	*The 13th Five-year Plan for Healthy Aging (2016–2020)*	National Health and Family Planning Commission.etc	2017	To build smart health and caring service platform, connect medical and caring service providers at all levels to provide all kinds of health and caring services for the elderly
	*Action Plan for the Development of Smart Health and Elderly Care Service Industry (2017–2020)*	Ministry of Industry and Information Technology, Ministry of Civil Affairs	2017	To clarify overall planning for the development of smart home for elderly care, put forward specific action plans on key issues such as technology research and service promotion

### Characteristics of smart home for elderly Care in China

#### A policy-driven product rather than a technology-driven or demand-driven one

The rapid development of new technologies in Western countries brings about emergence of smart home for elderly care. On the one hand, smart home for elderly care is the product of technological progresses [18]. On the other hand, it meets the real demands of the elderly in contemporary society. Compared with young people, the elderly are physically and psychologically vulnerable. Smart home for elderly care is a good response to this challenge and could meet the demands of the elderly for independent living [19]. In contrast, the development of smart home for elderly care in China is a policy- driven product [20]. In the initial stage, the intelligent technology was used to better meet the demands of the elderly, but the development speed was slow. In response to this problem, China issued a series of policy documents such as *Instruction to Promoting Internet plus Plan, Action Plan for the Development of Smart Health and Elderly Care Services (2017–2020)* and so on. Since then, pilot programs for smart home for elderly care began to develop rapidly across the country. It is clear that the national policies play a decisive role in the rapid development of China’s smart home for elderly care. Although the policy-driven characteristic of China’s smart home for elderly care has a number of positive aspects, it also brought about some side effects.

China’s smart home for the elderly care is a policy driven product rather than a demand-driven one, which brings an immediate disbenefit that a large number of the elderly do not have good understanding of smart homecare. And thus, the elderly’s demand for smart homecare is at low level. In general, the elderly are sensitive to the price of intelligent products [21], and have high requirements for the convenience of intelligent devices [22]. Thus, their self-efficacy perspective on the use of intelligent technologies are relatively low [23]. In addition, they have difficulty in conceptualizing how intelligent technologies could actually contribute to their lives [24]. These factors negatively affect the elderly’s general cognition of and demand for smart home for elderly care in China. A social survey showed that most of elderly people in China have poor knowledge of smart care services, and only 27% of the elderly are familiar with smart home for elderly care [25]. It is very difficult for them to imagine the expected benefits of smart home care [26].

Since China’s smart home for elderly care is a policy-driven product rather than the product of technological progress, the technological foundation of China’s smart home is not quite stable, which are mainly manifested in the following two aspects. First, most enterprises carrying out smart home for elderly care can only provide the elderly with simple services such as caring service order, emergency rescue and simple online health advice, but they can hardly provide complex services such as health management, comprehensive assistance, and home security [27]. Second, there is no industry technical standard of smart home for elderly care in China. Each enterprise has its own service platform, and smart home care service provided by the enterprise only fit its own platform. In consequence, the smart devices and services form different platforms are not compatible with each other. This is not only unfavorable for the development of the smart home for elderly care, but also lead to other negative consequences.

#### Quasi-public goods rather than private goods

Smart home for elderly care in Western countries is a kind of commercial service, which is regarded as private goods. However, China’s smart home for elderly care is not only private goods, but also public goods. In other words, it is more like quasi-public goods. In the early time, home-based elderly care service in China was purely public goods provided by the government [28]. In 2006, State Council of China promulgated *Opinions on Accelerating the Development of Elderly Care Services*, which aimed to flourish the consumption market of elderly care service. In 2010, the General Office of the State Council issued *Construction Plan of Social Elderly Care Service System (2011–2015)*, which proposed that China would give full play to the basic role of market in the allocation of home-based elderly care services. *The 12th Five-year Plan for China’s Undertakings for the Aged* issued by the State Council in 2011 aimed to strengthen the development of the ageing industry. It is clear that the government plays a major role in developing elderly care services. Therefore, home-based elderly care services in China is quasi-public goods, which share the dual attributes of public goods and private goods. This quasi-public goods’s attribute is also held by smart home for elderly care.

The quasi-public goods’s characteristic of China’s smart home for elderly care has dual development goals, that is, industry development and welfare progress. The goal of industry development emphasizes the attributes of the private goods underlying smart home care, which advocates that smart home for elderly care services should aim at making profits and be committed to expanding the consumer market. However, the goal of welfare progress lays more emphasis on the attributes of the public goods. It advocates that the purpose of smart home for elderly care should improve social welfare and provide the elderly with high-quality and low-cost care services. As a socialist country, China has a strong tradition of collectivism where people expect the government to provide elderly care services as public goods. However, as a developing country, China is unable to provide completely free care services for the elderly with limited fiscal revenue. As a consequence, there is a certain degree of opposition between the goals of industry development and welfare progress, which brings many challenges for the development of smart home for elderly care in China [29].

The opposition between the two developmental goals of China’ s smart home for elderly care at the macro level also leads to the contradiction between supply and demand at the micro level. Elderly people as the demand side expect low-cost or even free smart home for elderly care, while enterprises as the supply side hope to provide paid services to make profits by offering smart home care services. According to a survey in Qingdao, the average price of service of laundry, cleaning and accompanying care provided by Qingdao Smart Home Care Platform is 23.3 yuan, 28.6 yuan and 24.5 yuan per hour respectively, which exceeds the demand price of 10.3 yuan,14.2 yuan and 17.3 yuan per hour. The elderly generally regard smart home care services provided by the government platform as public goods and should be lower in price than other commercial services [30]. However, the prices of the most care services provided by government platform are close to those provided by private aged care providers such as 58.com and Ganji.com.

### Challenges to smart home for elderly Care in China

The unique characteristics of China’s smart home for elderly care have brought unique challenges for its development. We will firstly state the unique challenges faced by China’s smart home for elderly care, and then analyze the causes of these challenges. Finally, we characterize the causative relationships between the characteristics and challenges of smart home for the elderly in China.

#### Insufficient demand for smart home care caused by poor social perception and multiple stakeholders

The elderly in China hold poor perceptions of smart home care, which directly leads to the insufficient demand for smart home care services. A field study on the elderly in Qingdao found that 93% of respondents had never heard of smart home care. However, when they have a good understanding of the characteristics of smart home care service, 63% of the respondents believe that smart home care service can help them live a better life, which indicates that the elderly’s perception of smart home care service in China needs to be strengthened [30]. At the same time, most of the Chinese elderly regard smart home care service as a welfare product. In contrast, the care service providers regard smart home care service as commodity, and hope to make profits through service supply. Thus, smart home care services tend to be expensive. At present, the elderly have no habits of consuming expensive products and caring services [31]. Thus, it is difficult to popularize smart home for the elderly in China.

Insufficient demand for smart home care also bring about insufficient supply in the service market. There are a small number of users for many smart home care service platforms. It is thus difficult for the service providers to cover their cost, which leads to their survival dilemma. As a consequence, many enterprises are not willing to participate in smart home care services though they could be funded by the government. A field survey in Qingdao found that a large number of elderly care service providers, such as Zhongkang Love Neighborhood, Qingdao Lanchuang Technology, Qingdao Huakai, have troubles in sustaining their smart home care services due to the insufficient demand [30].

#### Disorderly development caused by lack of regulation on standard and quality

The lack of industry technical standards for China’s smart home care services has led to its disorderly development. Technical compatibility has been an important factor, which limits the popularization of smart home care in many countries [32]. It also occurs in China, where a unified technical standard of smart home care service has not been achieved both at national level and city level. Taking Qingdao, a major city in China, for an example, it has not established a unified technical standard among various smart home care platforms. in addition to the official Qingdao smart home care Platform (www.qingdaoyanglao.com), there are a number of smart elderly care service platforms including Zhongkang Love Neighborhood, Qingdao Lanchuang Technology, Qingdao Huakai and so on. Each platform has its own equipment and technical standards. Correspondingly, products and services provided by different smart home care platforms are incompatible with each other. This would lead to the disorderly development of smart home care in China.

In addition, there is no national regulation on the quality of smart home care, which restricts the development of service quality and the formation of a competitive market [33]. At present, multiple government departments are jointly responsible for the quality supervision over smart home care. For instance, Ministry of Civil Affairs is responsible for the quality supervision over smart daily care service provided by the government; State Administration for Market Regulation is responsible for the quality supervision over smart home care in the market; Ministry of Industry and Information Technology (MIIT) is responsible for regulating the operation of smart home care platform and the smart security service. At present, departments and agencies involved in managing smart home services have not coordinated with each other to issue a national quality regulation for smart home care. Given the complexity of smart home care, there still is a long way to go before a national quality regulation could be issued.

#### Waste of public and private resources caused by development surpass current stage

In many cities, smart home care platforms have not provided care services for the elderly, which results in a waste of public resources. In response to the national proposal of the application of “Internet plus” plan to the elderly care services, many local governments across China has established smart elderly care service platforms. However, in the case of the insufficient demand for smart home care services, a number of smart home care platforms funded by the government do not really work, which resulted in a waste of public resources. In fact, the development of smart home for the elderly is a gradual process, which needs the economic, social, cultural and technological support. A large-scale pilot projects concerning smart home care platform were established when the relevant social conditions are not generally favorable. In this sense, the market demand for smart home care services is now very low.

**Fig. 1:**
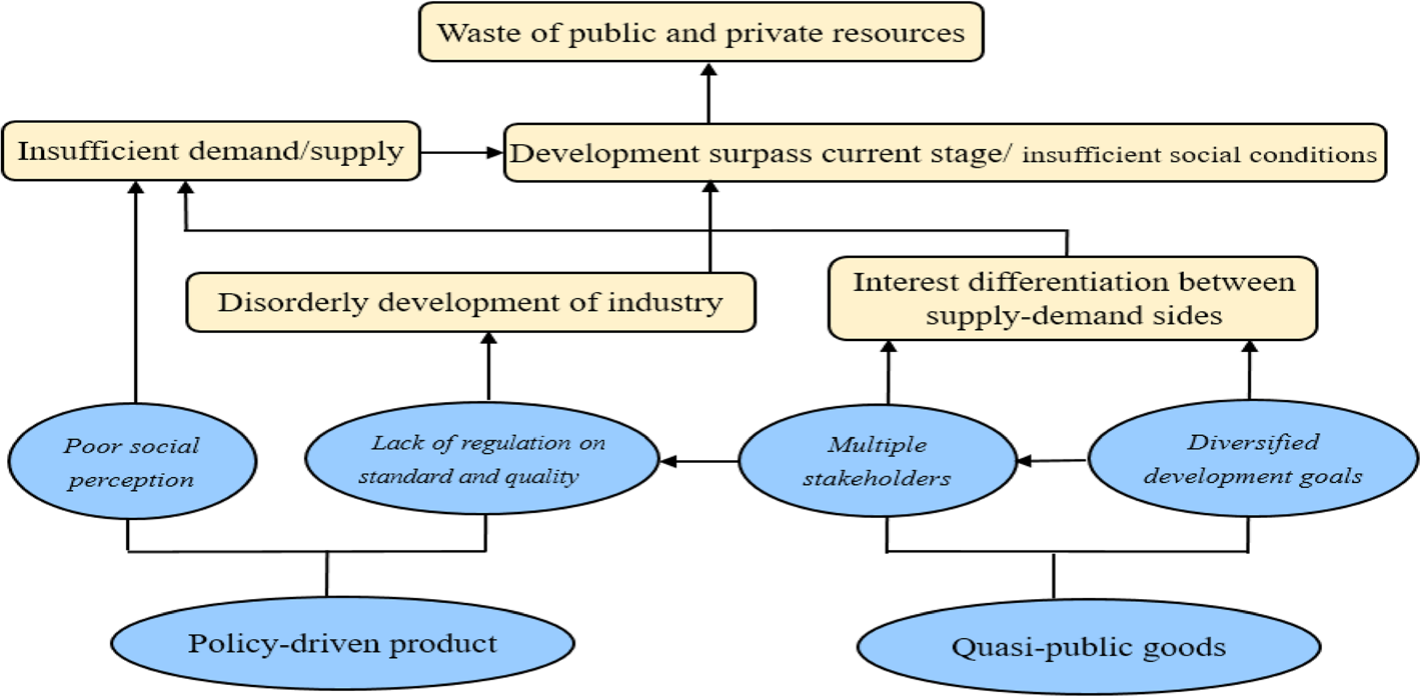
Main challenges and their reasons of China’s smart Home for elderly Care

According to the analysis above, the unique characteristics of smart home for the elderly are the main challenges for its development. To make the result clearer, we characterize the causative relationships between the characteristics and challenges of smart home for the elderly in China, which is shown in Fig. 1.

## Conclusions

### Prospect of China’s smart home for elderly care

At present, the family structure change, the immigration of young people, anticipated increases in chronic disease burden, and possible attenuation of filial care lead to an increasing demand for home-based care in China. It seems to provide a good opportunity for the rapid development of smart home care services in China. However, the popularization of smart home for elderly care is still a challenge for China. Its cost-benefit ratio should be improved, the elder’s willingness should be further inspired, and the incompatibility of services and products provided by different smart home care should be overcame.

In the process of theoretical research, scientific connotation and boundary of smart home for elderly care are basically clarified, and abundant reserves in concepts and technologies of smart home for elderly care are realized, thus providing a solid technical basis for the development of smart home for elderly care people. It has been shown that smart home for elderly care can promote the physical and mental health of the elderly, maintain their independent living, and improve their quality of life [34–36]. With the rapid development of science and technology, the cost of smart home for elderly care equipment and services will be greatly reduced, which makes smart home for elderly care available to more and more elderly people. In addition, many local industry associations are making efforts to set up industry technical standards, which will bring the possibility of platforms’ opening up between each other and thus promote the rapid popularization of smart home for elderly care [37].

Therefore, China’s smart home for elderly care has a bright future, which in turn will contribute to the development of global smart home for elderly care.

### Policy suggestions for China’s smart home for elderly care

Although great progress has been made in China’s smart home care, much efforts are still needed to further advance its development. Generally, China’s smart home for elderly care should focus on the following aspects. First, the technical standards for the elderly care services should be formulated as soon as possible and the existing public and private smart home for elderly care platforms should be combined. The establishment of industrial technical standards can prevent the disorderly development of the smart home for elderly care and avoid the incompatibility caused by product heterogeneity. The combination of the public and private smart care platforms can optimize resource allocation and management and avoid the waste of public resources. Second, enterprises should be encouraged to develop new technologies to reduce the cost of products and services provided by smart home for elderly care. Enterprises should take the initiative to achieve technical breakthroughs on the basis of the industry standardization and finally make the price of smart home for elderly care could be acceptable by more and more elderly people. Third, enterprises involved in offering smart home care services should implement customer-oriented strategy. At present, disabled and elderly people have great demand for smart care services, and thus these groups could become the main target market of smart home for elderly care. Enterprises should develop more products and services suitable for disabled and elderly people. Generally, smart home in China and in other countries provides people with various types of services, including health services, safety services, auxiliary services and nursing services [38]. These services could contribute a lot to the independent living of the elderly and improve their quality of life significantly.

## Data Availability

Not applicable.

## Abbreviations

IoTs: Internet of Things
ICT: Information and Communication Technology
AAL: Ambient Assisted Living
NDRC: National Development and Reform Commission
CNWCA: China National Working Commission on Ageing
NHFPC: National Health and Family Planning Commission
MIIT: Ministry of Industry and Information Technology

## References

[1] Chomik R, Piggott J (2015). Population ageing and social security in Asia. Asian Economic Policy Review.

[2] European Commission. The 2012 Ageing Report: Economic and budgetary projections for the 27 EU Member States (2010–2060). Available at: http://citeseerx.ist.psu.edu/viewdoc/download;jsessionid=178782C46AA694FC8121D42A3197E78A?doi=10.1.1.397.2801&rep=rep1&type=pdf [Accessed 27 Apr 2020].

[3] Sparrow R, Sparrow L (2006). In the hands of machines? The future of aged care. Minds & Machines.

[4] Beard J (2010). Innovative approaches to dealing with population ageing. Gerontechnology..

[5] Shinkle D, Tassin L (2011). Aging in place: a state survey of livability policies and practices. Saturday Evening Post.

[6] Peine A, Rollwagen I, Neven L (2014). The rise of the “innosumer”- rethinking older technology users. Technological Forecasting & Social Change.

[7] Harper R (2003). Inside the smart home.

[8] Silva L, Morikawa C, Petra I (2012). State of the art of smart homes. Eng Appl Artif Intell.

[9] Junestrand S, Tollmar K (1999). Video mediated communication for domestic environments - architectural and technological design. Lect Notes Comput Sci.

[10] Alaa M, Zaidan A, Zaidan B (2017). A review of smart home applications based on internet of things. Journal of Network & Computer Applications.

[11] Demiris G, Rantz M, Aud M (2004). Older adults’ attitudes towards and perceptions of ‘smart home’ technologies: a pilot study. Medical Informatics & the Internet in Medicine.

[12] Åkerlind C, Martin L, Gustafsson C (2019). Care managers’ perceptions of e-homecare: a qualitative interview study. Eur J Soc Work.

[13] Sabina M, Sabina M (2015). AAL: ambient assisted living assistive technologies for healthy ageing and opportunities for medicine and caring. Therapeutische Umschau Revue thérapeutique.

[14] State Council Information Office of People’s republic of China. Available at: http://www.scio.gov.cn/32344/32345/33969/34729/xgzc34735/Document/1481612/1481612.htm [Accessed 25 Apr 2020].

[15] Xi H, Ren X, Zhai S (2014). Smart homecare: the elderly care service innovation with information technology. Scientific Research on Aging.

[16] Wang Z (2017). National policies and measures to promote smart home for elderly care. China Social Work.

[17] Li S (2016). Shanghai has explored and taken measures to build the smart home for elderly care service system under the background of “internet plus” plan. Hum Resour Manag.

[18] Donald M, Judy W. The social shaping of technology – 2ed edition. Buckingham: Open University Press; 1999.

[19] Cheek P, Nikpour L, Nowlin HD (2005). Aging well with smart technology. Nurs Adm Q.

[20] Geng Y, Wang H (2017). Research on the development of internet plus service for the aged: transformation, integration and new commercial activities. Journal of Tianjin University of Administration.

[21] Lee C (2013). Adoption of smart technology among older adults: challenges and issues. Public Policy & Aging Report.

[22] Murata A, Iwase H (2005). Usability of touch-panel interfaces for older adults. Hum Factors.

[23] Czaja SJ, Charness N, Fisk AD (2006). Factors predicting the use of technology: findings from the center for research and education on aging and technology enhancement (create). Psychol Aging.

[24] Walsh K, Callan A (2011). Perceptions, preferences, and acceptance of information and communication technologies in older-adult community care settings in Ireland: a case-study and ranked-care program analysis. Ageing Int.

[25] Gao L, Liu D, Song S (2019). Analysis of influencing factors of community elderly’s demand on smart home care. Health Vocational Education.

[26] Mao Y, Li D (2015). A study on factors influencing the use behavior of smart home for elderly care based on UTAUT model: a case study of Wuhan. E-government..

[27] Xu X (2019). Resource shortage or resource dependence: resource dilemma of elderly care service in smart community. Lanzhou Academic J.

[28] Guo J (2010). International comparison and reference on home pension service mode. Social Security Res.

[29] Jiang C (2016). Smart endowment ecological chain construction under new normal: based on supply and demand perspective. J Shandong Univ Finance Econ.

[30] Zhang Q, Li L, Ji G (2018). Challenges and solutions to the coordinated development of smart home for elderly care in Qingdao. Outstanding research compilation of human resources and social security department of Shandong province, China.

[31] Geng Y, Wang X (2017). The internet plus service for the elderly: opportunities, predicaments and solutions. J Shenzhen University (Humanities and Social Sciences Edition).

[32] Mynatt ED, Rogers WA (2001). Developing technology to support the functional independence of older adults. Ageing Int.

[33] Zhang C, Li J (2018). Research on the current development and standardization of smart homecare home and abroad. China Standardization.

[34] Neven L (2010). 'But obviously not for me': robots, laboratories and the defiant identity of elder test users. Sociol Health Ill.

[35] Cotten S, Ford G, Ford S (2012). Internet use and depression among older adults. Comput Hum Behav.

[36] Siegel C, Hochgatterer A, Dorner TE (2014). Contributions of ambient assisted living for health and quality of life in the elderly and care services-a qualitative analysis from the experts’ perspective of care service professionals. BMC Geriatr.

[37] Peine A (2009). Understanding the dynamics of technological configurations: a conceptual framework and the case of smart homes. Technol Forecasting Soc Change..

[38] Zhang Q, Li H (2019). From possibility to feasibility: international research progress of smart home for the elderly people and enlightenment for China. Learning and Practice.

